# N P Singh: History of the first intensive care unit in Delhi – reminiscences

**DOI:** 10.4103/0019-5049.72652

**Published:** 2010

**Authors:** SP Devanandan

**Affiliations:** C.M.C., Vellore - 632 002, Tamil Nadu, India

## Abstract

As most of us are aware, ventilator support came to stay after the polio epidemic in Denmark in the ‘50s. Many of us are also aware that Peter Safar, an Anaesthesiologist, is credited with pioneering cardiopulmonary resuscitation (CPR), who also wrote a book titled “The ABC of Resuscitation” in 1957 for training the public in CPR. It was later adopted by the American Heart Association. He also started the first intensive care unit (ICU) in 1958 in the USA. Ten years later, from 1968, the specialty grew from strength to strength in our country and, in 1992, the Society of Critical Care Medicine was formed.

In 1968, going back 40 and odd years, my colleagues and I were registrars in the Department of Anaesthesia at the Maulana Azad Medical College and associated Irwin and Pant Hospitals. Irwin Hospital, now known as Lok Nayak Jaya Prakash Narayan Hospital, was the largest hospital in Delhi, with more than 1,500 sanctioned beds, and many more on the floor. The Head of the Department of Anaesthesia was Prof. N P Singh. At the time that I worked with him, he was keen to get an ICU started in the Irwin Hospital. He met resistance at every stage – the Administration of the Hospital, Delhi Administration, the Ministry of Health and even from his senior colleagues at Irwin Hospital. It was not easy to get an appointment with the powers that be.

By a stroke of luck, he discovered that the Lt. Governor of Delhi was to inaugurate a Road Safety Week Exhibition conducted by the Chief of the Traffic Police. He took two of his staff (suitably kitted out) and went to the venue of the exhibition. Cornering the Chief of the Traffic Police, he told him that he came to see what all the activity was about and was all praises for the concept of public awareness of road safety. He described how Irwin Hospital dealt with 100s of accidents and what it cost the Government. Educating the public to obey traffic signals and avoid drinking and driving was important he said, but it could not totally prevent an accident (although, of course, it would reduce the number). In a matter of few minutes, he convinced the Traffic Chief that public awareness should be created on an “immediate, on the spot action” in the event of an accident and that this exhibition needed that one element for its complete success. The Chief was enthusiastic about the concept, but reluctantly admitted that his staff knew nothing about resuscitation. The Professor declared that he had come to cover this lacuna and make the Police Exhibition a grand success.

He was allotted a 10’×10’ stall for display of posters and equipment. The police provided furniture and stationary and gratefully gave the anaesthetic team a royal salute! On the day of the inauguration, the stall looked impressive. Doctors were costumed in their coats and stethescopes, with important-looking badges; there were drip stands and IV bottles and other equipment to grip the imagination and attention of the spectators. A complete full-length skeleton graced the stall, with two bags occupying the rib cage. The bags were ventilated by a Radcliffe ventilator of World War 2 vintage. Needless to say, for the wide-eyed public, this was far more interesting than the red, green and amber traffic lights and zebra crossings put up by the Traffic Police!

When the Lt. Governor came to inaugurate the exhibition and cut the ribbon, Prof. N P Singh was right in front to greet him and deftly led him to the resuscitation stall! A nonstop lecture-demonstration followed on all aspects of first aid – basic life support (BLS), advanced life support (ALS) and ambulance service. He stressed on the fact that because accidents could not be avoided, there was an urgent and immediate need for a resuscitation ward/ICU in what was one of the premiere institutions in the capital. The Lt. Governor, who was the ultimate authority to approve major changes, sanction major equipment and create new posts for medics and paramedics in Irwin Hospital, was suitably impressed. Very importantly, the press reporters who had come to cover the event were cornered and lectured on first aid, resuscitation and much more! The next day, the press reports on the inauguration of the Road Safety Week Exhibition looked and sounded like a plea for both educating the public in first aid and the acute need for a resuscitation ward/ICU in the largest hospital in Delhi, the Irwin Hospital.

After impressing the Lt. Governor of Delhi, Prof. N P Singh shifted his activity to Irwin Hospital. He literally “grabbed” a location near the Emergency Area (a strategic place adjacent to Casualty, Emergency OR and the Emergency Ward); equipment was procured by diverting funds allocated to other departments with the blessing of the Delhi Administration (and much to the anger of the affected departments). The day of inauguration of the resuscitation ward/ICU finally arrived, with junior doctors combing the entire hospital for patients to fill the beds. Finally, one patient was found who was waiting to be discharged after an overdose of barbiturate tablets. He, along with his case sheet, were transferred to the resuscitation ward/ICU. He was then instructed to lie still with his eyes closed. He played his part well! After the VIPs had departed, Prof. N P Singh returned to the ICU to retrieve his bag and found his entire staff along with the erstwhile “comatose” patient celebrating the occasion with Coca Cola. Being a good sport, he enjoyed the scenario.

Prof. N P Singh was born on 15.07.1931 and died on 04.12.2006. Although his obituary did mention the various professorial appointments he held, and the fact that he had organized 50 ambulances for the city of Delhi, I believe that he went to his grave “Unwept, Unhonoured and Unsung.” Being an MD (Anaesthesia), post-graduate student at Maulana Azad Medical College and affiliated Hospitals at a time when an ICU was being started was interesting, stimulating and hilarious, thanks to Prof. N P Singh.

I wish to point out here that, in 1968, the going was rough, even in the capital city of Delhi. It was the passion and persistence of one man – Prof. N P Singh – that brought about the advancement of resuscitation and intensive care in Delhi.

